# Common Marmosets: A Potential Translational Animal Model of Juvenile Depression

**DOI:** 10.3389/fpsyt.2017.00175

**Published:** 2017-09-21

**Authors:** Nicole Leite Galvão-Coelho, Ana Cecília de Menezes Galvão, Flávia Santos da Silva, Maria Bernardete Cordeiro de Sousa

**Affiliations:** ^1^Laboratory of Hormone Measurement, Department of Physiology, Federal University of Rio Grande do Norte, Natal, Brazil; ^2^Postgraduate Program in Psychobiology, Federal University of Rio Grande do Norte, Natal, Brazil; ^3^National Institute of Science and Technology in Translational Medicine Natal, Natal, Brazil; ^4^Brain Institute, Federal University of Rio Grande do Norte, Natal, Brazil

**Keywords:** behaviors, cortisol, chronic stress, early-age depression, non-human primate, translational animal model

## Abstract

Major depression is a psychiatric disorder with high prevalence in the general population, with increasing expression in adolescence, about 14% in young people. Frequently, it presents as a chronic condition, showing no remission even after several pharmacological treatments and persisting in adult life. Therefore, distinct protocols and animal models have been developed to increase the understanding of this disease or search for new therapies. To this end, this study investigated the effects of chronic social isolation and the potential antidepressant action of nortriptyline in juvenile *Callithrix jacchus* males and females by monitoring fecal cortisol, body weight, and behavioral parameters and searching for biomarkers and a protocol for inducing depression. The purpose was to validate this species and protocol as a translational model of juvenile depression, addressing all domain criteria of validation: etiologic, face, functional, predictive, inter-relational, evolutionary, and population. In both sexes and both protocols (IDS and DPT), we observed a significant reduction in cortisol levels in the last phase of social isolation, concomitant with increases in autogrooming, stereotyped and anxiety behaviors, and the presence of anhedonia. The alterations induced by chronic social isolation are characteristic of the depressive state in non-human primates and/or in humans, and were reversed in large part by treatment with an antidepressant drug (nortriptyline). Therefore, these results indicate *C. jacchus* as a potential translational model of juvenile depression by addressing all criteria of validation.

## Introduction

Major depression is a mood disorder, which is ranked as the most prevalent disease in the population. According to the World Health Organization (1), the estimated number of people with depression globally is over 300 million. Moreover, depression is ranked as the largest contributor to disability in the world (1, 2).

The symptoms of depression are expressed in varied and sometimes opposing ways: sleep, feeding and body weight alterations, fatigue, irritability, depressed mood, loss of interest, or pleasure in almost all daily activities, accompanied by feelings of guilt. In addition, psychomotor disorders can occur, although they are less common, and they are an indicator of severity in the individual’s situation (*DSM-5*). These symptoms are associated with neuroendocrine modifications and in severe cases, can lead to death by suicide (1–3).

Despite substantial progress in the understanding of depression and in the development of new antidepressants drugs, fewer than half of cases achieve clinical recognition, that is, are diagnosed. In diagnosed people, approximately half are treated, the same proportion receives adequate treatment, and 65% of this group achieves remission after satisfactory treatment (4, 5). Even after many systematic treatments, approximately one-third of patients do not achieve remission (6).

Some studies suggest that pharmacological resistance to the treatment, observed in a substantial number of patients, could be in part due to the use of inadequate protocols and/or animal models to investigate depression and to test antidepressants, demonstrating the need of different approaches (7). Rodents are commonly used to study depression and pharmacological antidepressant drugs due to the easy handling, possibility of using transgenic animals, and the presence of well-established protocols (2, 8, 9). However, several species and numerous stress protocols have been tested to develop other translational models that mimic the physiological and behavioral states observed in human depression (2, 10, 11).

Nevertheless, the validity of the model, that is, its consistency and predictive value, needs to be taken into account (12). In principle, in psychiatric studies, four criteria need to be considered when validating an animal model. First, regarding etiologic criteria, the model should develop the disease by the same cause or agent that induces it in humans. Second, the model should address the face criteria, which correspond to the ability of the proposed model to present behavioral and other symptoms observed in humans. Third, the functional or content criteria involve the capacity of the model to show similar physiological alterations to those observed in human patients. The last criteria include the predictive value, which is the reversion of these symptoms by effective pharmacological treatments used in humans (7, 13, 14).

Therefore, in practice, only face and predictive criteria are considered in the studies. The induction of a state of depression in animal models is accomplished by protocols applying acute physical stressors such as restraint, food or water restriction, continuous light exposure, physical or chemical lesions or genetic knockout (14–16) or social stressor paradigms such as social defeat (17) and early social separation (18), whereas in humans, chronic psychosocial stress is typically the agent of onset of this disease (19). In addition, the protocols used in rodents to investigate depressive state sometimes do not have etiological validity, i.e., the forced swimming (20) test normally used in studies of depression is not a relevant stressful situation for all species of rodents in nature, and exhibits low ecologic validity for some species (15, 21). Therefore, the etiologic criteria are not addressed in various studies, and this is a cause for concern, because the face aspects observed are not likely an evolutionary trace. Moreover, investigations with molecular biomarkers such as hormones, neurotransmitters, or cytokines associated with behaviors are scarce in animal models of depression (22); therefore, the content criteria are also not addressed in most studies (15, 22–24).

More recently, to improve studies conducted in animal models, other criteria have been considered to validate translational models in psychiatry: inter-relational, evolutionary, and population (25). Inter-relational criteria propose to investigate a model in various and interacting disordered domains such as behaviors, molecular biomarkers, and cognition (25). Evolutionary criteria reflect the ability of the proposed model to investigate determinate disordered domains in a similar manner across various species. In depression disorders, feeding, somnolence, motor alteration, and anhedonic behaviors, as well as cortisol levels and body weight, are altered in humans, non-human primates, rodents, and other species and can be used to investigate evolutionary criteria. Therefore, these indicate conserved phenomena in this pathology along with evolution with great importance placed on its understanding, mainly because it can be associated with subjective states that cannot be measured directly in animals (12, 25, 26). The population validity criterion is the capacity of the proposed model to reflect the natural variance in phenotypes observed in the general population. The use of inbred or knockout strains to reduce random noise in studies also reduces the variability and does not reflect the heterogeneity of the human population. In this context, it is better to use outbred or wild-type populations (25).

The brain morphology, neural functional organization, social organization, and evolutionary history of non-human primates are more closely related to those of humans than are those of other species commonly used as animal models of depression, such as rodents and fish (27, 28). Consequently, the use of non-human primates as animal models in studies of depression addresses both traditional and newly proposed validation criteria.

Moreover, it was recently observed that macaques exhibit naturally occurring depression attributed to chronic psychosocial stress, similar to that observed in humans. Consequently, in non-human primates, depending on their social organization, the stress protocols involving changes in social rank or chronic social isolation can induce similar physiological and behavioral symptoms to those observed in depressive disorders in humans (11, 28–30).

The majority of studies of mood disorders are developed in adult or infant animals and only a small proportion are conducted in juveniles. It is well known that juvenile age is an important ontogenetic phase because it is a biological window of plasticity of the nervous system, showing considerable susceptibility to environmental influences that might induce permanent changes in cognition and in the stress response system. If these changes are maladaptive, they can induce serious and permanent damage in cognitive, behavioral, and physiological parameters in adulthood, increasing the probability of the emergence of mood disorders such as depression (31–33). The incidence of depressive episodes in adolescents has increased (34), reaching 14% of the youth population aged between 15 and 18 years, with a recurrence rate of approximately 40% in 3–5 years following the first episode (31).

In this context, the present study investigated the effects of chronic social isolation and the potential antidepressant effect of nortriptyline on physiological parameters (fecal cortisol and body weight), as well as on the behavioral repertoire, in juvenile male and female common marmosets (*Callithrix jacchus*) to characterize biomarkers that respond to an etiological protocol for the study of depression in non-human primates. Thus, our intention is to validate this animal model and protocol with respect to distinct criteria including etiologic, face, functional, predictive, inter-relational, evolutionary, and population, in order to provide evidence for the utility of this species as a translational juvenile animal model of depression.

The common marmoset, *C. jacchus*, is a small and social non-human primate that adapts well to captivity and has a high fertility rate, when compared with Old World primates (35–37). This species exhibits a range of changes at the physiological and behavioral levels that resemble those observed in humans when facing stress (11, 38–40). *C. jacchus* also display complex social organization and a number of similar social behaviors to humans and alloparental care (41), making this species relevant for the use as a model in several areas of research, including affective disorders such as anxiety and depression (42, 43). Moreover, *C. jacchus* has a well-defined ethogram (44) and a non-invasive technique to measure steroid hormones (cortisol, progesterone, and androgens) in feces (45, 46), which facilitates its use as an experimental model in disorders associated with the hypothalamic–pituitary–adrenal axis (HPA), such as depression (38).

## Materials and Methods

### Study Design

This study includes two experimental procedures to validate the use of the common marmoset as an animal model of juvenile depression. The first protocol was performed to validate chronic social isolation as a protocol for induction of depression state (IDS) and to provide evidence validating the etiologic, face, and functional criteria of this depression animal model. To address the predictive criteria of validation, a second protocol was developed, namely, depression state + pharmacological treatment (DPT), to attempt to reverse the symptoms and physiologic alterations observed in isolated context (IC) by using antidepressants.

### Protocol for IDS

**Figure 1:**
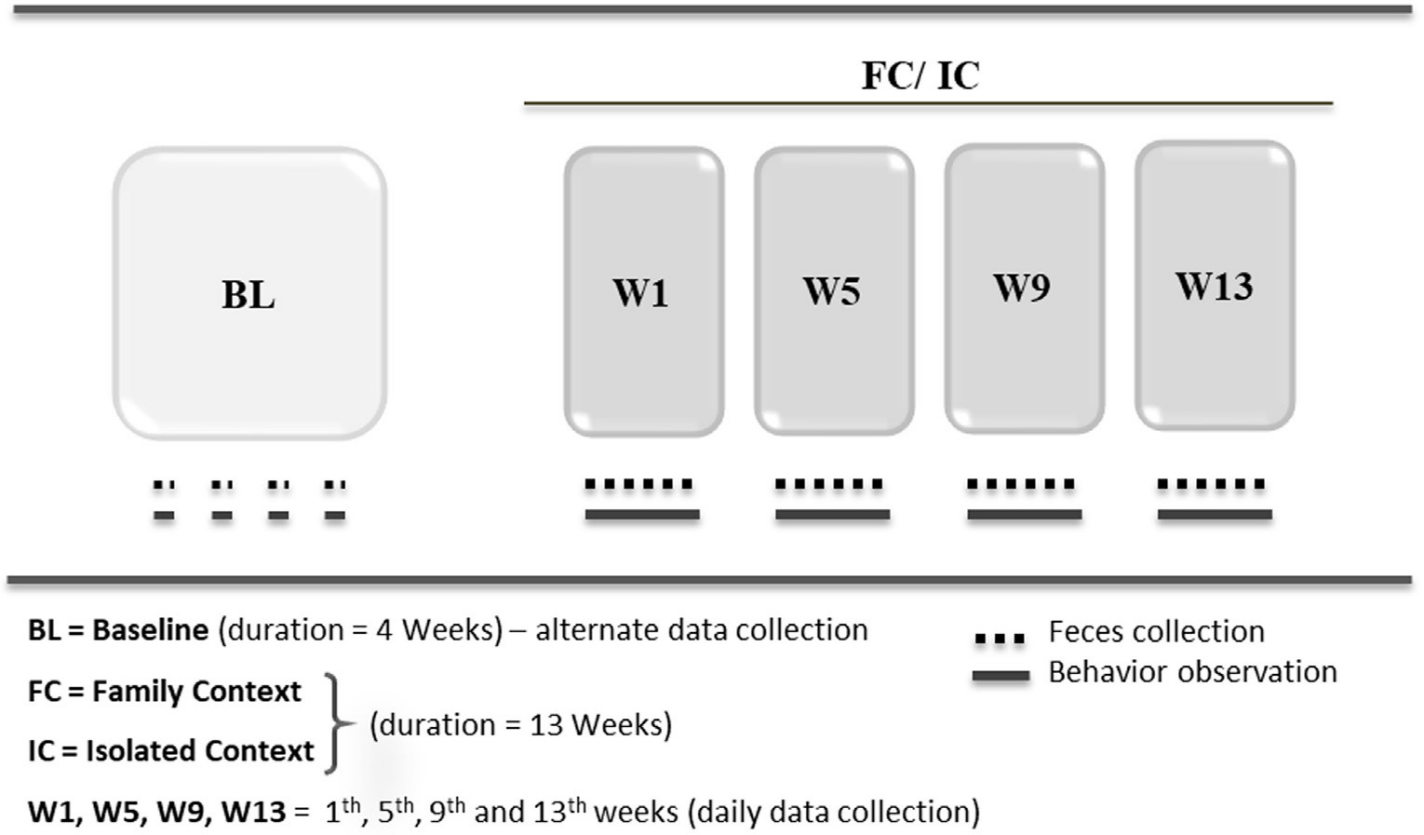
Protocol for induction of depression state (IDS).

This experimental procedure included two phases, and the objective of this protocol was to induce a state of depression using the paradigm of chronic social isolation in common marmoset juveniles (Figure 1):

(1) *Baseline (BL)*: ten juvenile males (according to the classification of age by Leão et al. (47)) of common marmosets, aged approximately 7 months, were monitored for 4 weeks living in cages with their families. Fecal samples for cortisol measurements and behavioral data were collected on alternate days to establish the hormonal and behavioral profiles of the animals of the animals.
(2) *Social context* [familiar context (FC)/ isolated context (IC)]: after BL, the animals were divided randomly in two groups with distinct social context and were monitored for 13 weeks: (a) FC—five juvenile males remained in their home cages with their respective families; (b) IC—five juvenile males were socially isolated, separated from their families, and placed alone in new cages.

During 13 weeks of social context, data collection (fecal sampling and behaviors) was performed daily in the 1^st^, 5^th^, 9^th^, and 13^th^ weeks (W1, W5, W9, and W13, respectively) for both groups (FC and IC). The duration of social isolation was determined according to similar studies of depression in non-human primates (48, 49).

### Protocol for DPT

**Figure 2:**
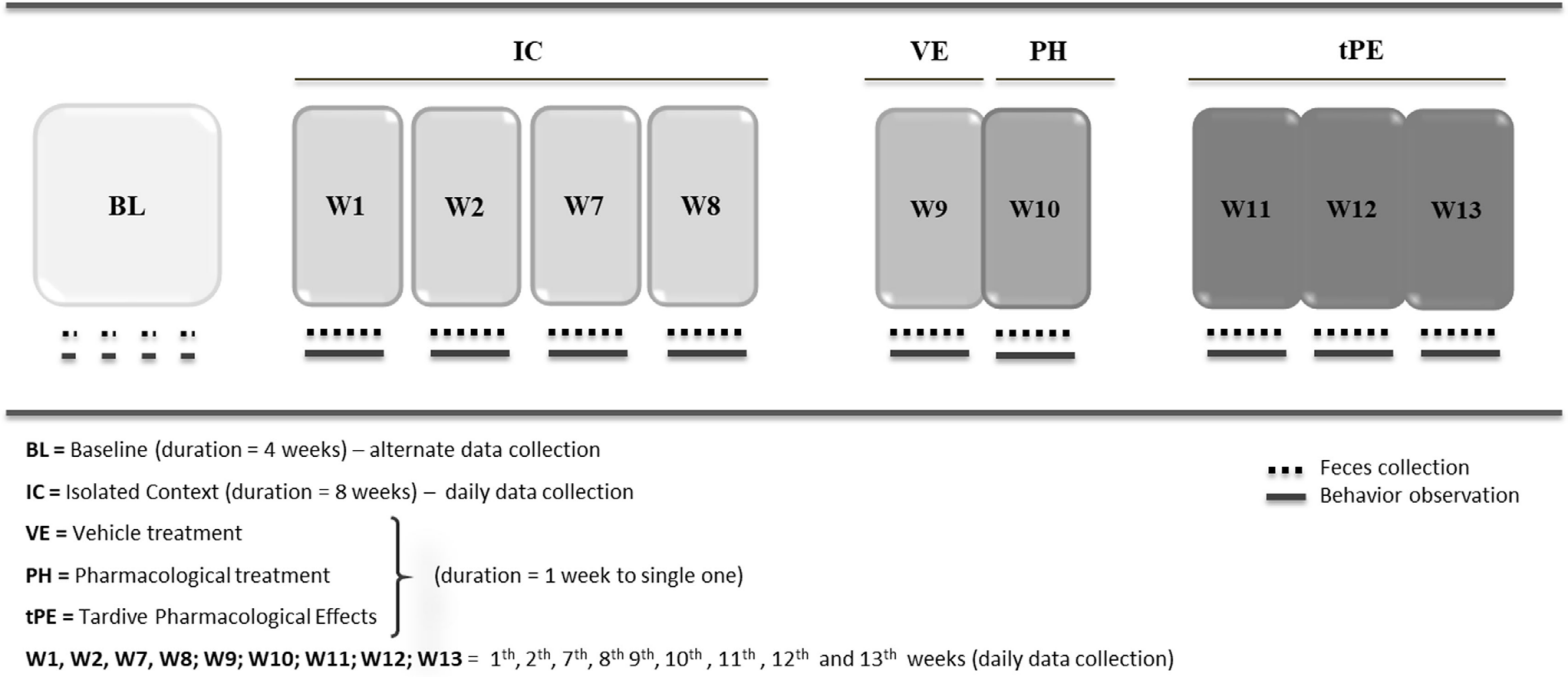
Protocol for depression state + pharmacological treatment (DPT).

The second experimental procedure included five phases (Figure 2). The objective was to evaluate whether the depressive state could be reversed by nortriptyline, an antidepressant drug commonly used in humans, to demonstrate whether this animal model adhered to the predictive validity criterion. Moreover, this protocol was developed to investigate whether sexual dimorphism occurred in the depressive state in this species and at this age. To that end, eight juvenile males and seven juvenile females of common marmosets were observed consecutively in four situations:

(1) *Baseline*: similarly to the IDS protocol, in the BL phase 15 juvenile common marmosets, 8 males and 7 females, aged approximately 7 months, were observed for 4 weeks living with their families. Fecal sampling for cortisol measurement and behavioral data collection were performed on alternate days to establish the hormonal and behavioral BL profiles of the animals.

After the BL period, all animals were sequentially monitored across four conditions:

(2) *Isolated context*: the animals were separated from their families and were socially isolated for 8 weeks in new cages. Under this condition, data collection (feces and behaviors) was performed daily in the first 2 weeks of the first month (W1 and W2) and in the last 2 weeks of the second month (W7 and W8, respectively).
(3) *Vehicle (VE) treatment*: after the eighth week of isolation, three males and three females were randomly selected to be treated with saline solution as a VE, prepared using 9.8 mL of saline mixture to 0.2 mL of Tween 80. All animals received one daily intraperitoneal administration of saline (0.2 mL/100 g animal), for 7 days in week 9 (W9). Behavioral and fecal data collections were performed daily.
(4) *Pharmacological treatment* (PH): following the VE, the same animals received one daily intraperitoneal administration of nortriptyline hydrochloride (Pamelon Novartis) (12.5 mg/mL) for 7 days in week 10 (W10) at the same volume used for saline solution (0.2 mL/100 g of nortriptyline). The dose of nortriptyline was determined based on a study with rats, whereby the effective antidepressant dose was found to be approximately 30 mg/kg (50). The ip administration method was chosen after a number of unsuccessful attempts to induce the animal to ingest the drug together with several different food items. Similarly, for the previous condition, behavioral and fecal data collections were performed daily (29).
(5) Tardive pharmacological effects (tPEs): after PH, the same animals were monitored for a further 21 days to observe post-pharmacological symptoms. This period was divided into 3 weeks (week 11 = W11; week 12 = W12; week 13 = W13) to facilitate statistical analysis of the data. Fecal data collections were performed daily.

### Animal Maintenance

All 25 animals used in this study (IDS = 10 males and DPT = 7 males and 8 females) lived in captivity in the Laboratory of Advanced Studies in Primates of the Universidade Federal do Rio Grande do Norte (UFRN), Natal, Brazil. In order to address the population validity criteria and mimic the individual variability observed in the human population, the animals were randomly selected from 19 different families. In experiment 1, only juvenile males were available for experimental use in the primate colony. However, after validation of the stressor protocol in experiment 1 and considering the high prevalence of depression in females since the adolescence phase, and the fact that the colony was able to provide animals of both sexes, we developed the other experimental phases of the study using males and females.

In the BL periods, the animals lived with their families, in outdoor cages, under conditions of natural lighting, humidity, and temperature. The cages measured 2.0 m × 2.0 m × 1.0 m and were built of masonry. The front consisted of a glass wall with a unidirectional visor and on the back wall, a wire mesh door, on which were placed a water bottle and a plate of food. Inside the cage were placed a nest box in which to rest, planks of wood, and branches of plants for environmental enrichment and to allow the animals to move around the cage.

During the social isolation phases of both procedures (IDS and DPT), the animals were placed in new masonry cages with different dimensions (1.0 m × 2.0 m × 1.0 m) from those in which they were living in family groups, but without space restrictions. These new cages were also located outdoors. In this condition, the animals did not have any visual contact with conspecifics but had auditory and olfactory contact with other conspecifics that were not members of their own families.

None of the animals had been separated from their respective family groups for prolonged periods and were habituated to the presence of the researchers prior to the study. Veterinary care was provided throughout the experiment. Water was available without restriction during the entire study and all animals were fed twice a day with the same diet, which included seasonal fruits such as banana, papaya, melon, and mango, as well as potato and a protein potage containing milk, oats, egg, and bread. A multivitamin supplement (Glicocan) was administered twice a week.

In order to address the inter-relational validity criteria for validation of this translational animal model, the animals were weighed approximately every 15 days.

The animals were housed according to IBAMA (Brazilian Institute of Environment and Renewable Natural Resources) guidelines (Normative Instruction no. 169 of February 20, 2008), and the care standards for animals established by CONCEA—National Council for Animal Experimentation Control, Law No. 11.794 (October 8, 2008). In addition, the laboratory complies with international standards for *ex situ* maintenance of animals as defined by the Animal Behavior Society and the International Primatological Society. The study and experimental procedures were approved by the Animal Research Ethics Committee (UFRN protocol No. 019/2013 and protocol No. 034/2014).

### Behavioral Records

A continuous fecal sampling method was used to evaluate the frequency and/or duration of the selected behaviors. Recording was performed continuously for each animal over a 30-min period (51). Behavioral data were always collected between 06:30 and 07:30 a.m. to avoid the influence of circadian variation (52). Descriptions of behaviors were according to the ethogram compiled by Stevenson and Poole (42) (Table 1).

**Table 1:** Description of behaviors according to the ethogram compiled by Stevenson and Poole and in context of stress.

Behavior	Description	Context of stress
Scent marking	Act of scrubbing the anogenital and suprapubic region on a substrate	Expression of anxiety (53, 54)

Individual piloerection	Act of erection of the pelage and walking with an arched back	Expression of activation of the sympathetic nervous system (54)

Autogrooming	Act of self-grooming	Acts as a stress/tension reducer (55, 56)

Locomotion	Act of moving randomly between four different quadrants of the same size, delimited in a cage	Expression of anxiety (53, 54)

Scratching	Act of using hands to scrub some body region	It is considered a stereotyped behavior (29)

Somnolence	Characterized by a slow blink, sleepiness, and stare	

Feeding	Act of taking a piece of food to the mouth and ingesting it	

Ingestion of an aqueous solution of sucrose (4.16%)	Act of drinking a palatable substance (sucrose solution)	The reduction of this pleasurable behavior can express a possible state of anhedonia (29, 57)

Typical behaviors for common marmosets, which included scent marking (frequency), individual piloerection (frequency), scratching (duration), and autogrooming (duration) were recorded. Indeed, behaviors that can be compared across species to address evolutionary validity, such as locomotion (frequency), somnolence (duration, investigated only in DPT), feeding (frequency for IDS/frequency and duration for DPT), and anhedonia were also recorded. The frequency (for IDS and DPT) and duration (DPT) of ingestion of an aqueous solution of sucrose (4.16%) was measured to verify a possible state of anhedonia [adapted from Paul et al. (58)].

### Fecal Collection and Cortisol Assay

In order to address the functional and inter-relational criteria for validation of this translational animal model, feces were collected for the measurement of cortisol. Fecal collection was performed in the morning between 6:30 and 8:30 a.m. to avoid circadian variation in cortisol measurement (59). Fecal cortisol reflects plasma cortisol with a delay of approximately 8–10 h (45).

Prior to fecal collection, the cages were cleaned to facilitate sample identification. The observer entered the cage shortly after animal defecation and collected the sample with a clean wooden stick. Samples were identified and stored at approximately −4°C until the day when they were processed for cortisol extraction and quantification at the Hormonal Measurements Laboratory of Department of Physiology—UFRN, according to the protocol of Sousa and Ziegler (45). Intra- and inter-assay coefficients of variation for fecal cortisol obtained in this study were 1.78 and 15.47%, respectively, for samples collect during IDS and 2.74 and 16.61%, respectively, for samples collected during the DPT procedure.

### Statistical Analysis

Hormonal data were normalized by logarithmic transformation, and for both hormonal and behavioral data, the statistical technique of bootstrap resampling was applied to the multivariable analysis. For cortisol, one outlier four standard deviations above the mean was excluded from the analysis in W5 of IDS.

General linear models (GLM) Fisher’s *post hoc* test were used to investigate the variations of behaviors, cortisol, and body weight between groups (FC/IC) or sex (in DPT) throughout the study phases. Moreover, the parametric Student’s *t*-test and the non-parametric Mann–Whitney *U* and Wilcoxon’s sum-rank tests were used in some investigations to analyze hormonal and behavioral data.

The correlations between behaviors and hormones and between behaviors were investigated in combined phases by the Spearman correlation test.

Results were considered statistically significant at *p* ≤ 0.05 and 0.05 < *p* < 0.07 was considered to indicate a trend in all the tests.

## Results

### Protocol for IDS: Etiologic, Face, and Functional Validity

#### Hormonal Profile

**Figure 3:**
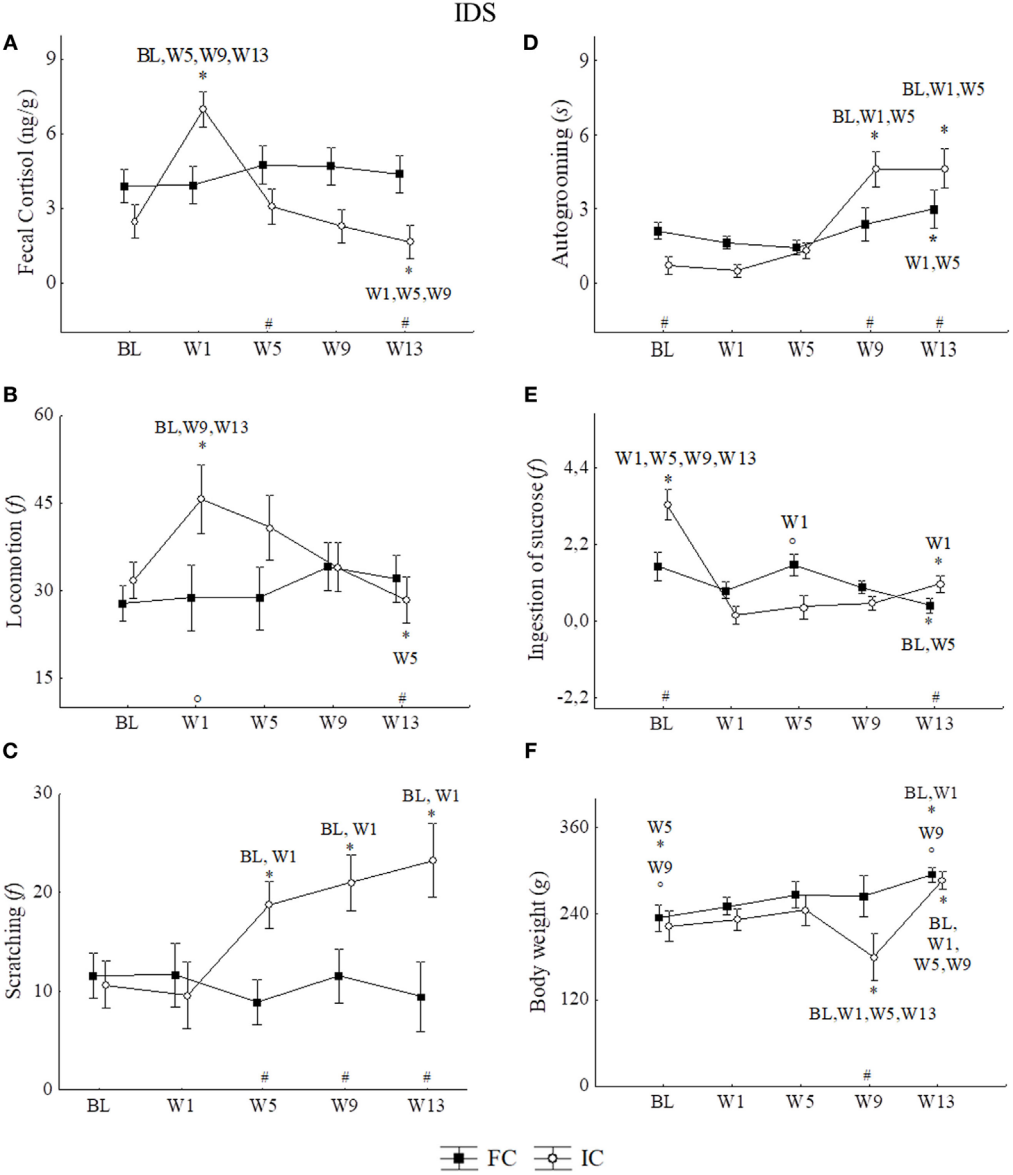
Mean of the week ± SEM for: **(A)** fecal cortisol, **(B)** locomotion, **(C)** scratching, **(D)** autogrooming, **(E)** ingestion of sucrose solution, and **(F)** body weight in the IDS (protocol for induction of depression state) for family context group (FC) and social isolated context group (IC) of male juveniles *Callithrix jacchus* in the baseline (BL) and weeks of the experimental protocol: 1st week (W1), 5th week (W5), 9th week (W9), and 13th week (W13). *Statistically significant (*p* ≤ 0.05) differences between the phase in which the symbol is and the (s) phase (s) indicated next to the symbol; ^#^statistical difference (*p* ≤ 0.05) between the groups in the respective phase; °statistically trend (0.05 < *p* < 0.07) of difference between the phase in which the symbol is and the phase indicated next to the symbol or between the groups in the respective phase. General linear models and *post hoc* Fisher.

No significant variation in cortisol levels was observed throughout the experiment in the FC group (*n* = 5). On the other hand, in IC (*n* = 5) cortisol levels were statistically higher in W1 than in BL, W5, W9, and W13. After W1, cortisol profiles showed a gradual reduction along the weeks, with lower values in W13 as illustrated in Figure 3A. All statistical values are in Table 2.

**Table 2:** Statistical values of the protocol for induction of depression state (IDS).

	Fecal cortisol					Autogrooming				Frequency of ingestion of sucrose solution				Body weight				Locomotion				Scratching			
	*F* = 3.92, *p* = 0.01, df = 4					*F* = 5.49, *p* = 0.01, df = 4				*F* = 8.16, *p* = 0.00, df = 4				*F* = 3.44, *p* = 0.02, df = 4				*F* = 2.35 *p* = 0.05, df = 4				*F* = 13.98, *p* = 0.01, df = 4			

	W1	W5	W9	W13	W1	W5	W9	W13	W1	W5	W9	W13	W1	W5	W9	W13	W1	W5	W9	W13	W1	W5	W9	W13
FC	BL	*p* = 0.77	*p* = 0.12	*p* = 0.74	*p* = 0.98	*p* = 0.47	*p* = 0.31	*p* = 0.71	*p* = 0.20	*p* = 0.08	*p* = 0.91	*p* = 0.14	*p* = 0.01	*p* = 0.32	*p* = 0.05	*p* = 0.07	*p* = 0.00	*p* = 0.87	*p* = 0.86	*p* = 0.25	*p* = 0.44	*p* = 0.86	*p* = 0.27	*p* = 0.59	*p* = 0.34
	W1		*p* = 0.20	*p* = 0.96	*p* = 0.76		*p* = 0.76	*p* = 0.28	*p* = 0.04		*p* = 0.06	*p* = 0.79	*p* = 0.27		*p* = 0.31	*p* = 0.38	*p* = 0.01		*p* = 0.99	*p* = 0.33	*p* = 0.55		*p* = 0.21	*p* = 0.71	*p* = 0.27
	W5			*p* = 0.22	*p* = 0.12			*p* = 0.17	*p* = 0.02			*p* = 0.11	*p* = 0.01			*p* = 0.88	*p* = 0.09			*p* = 0.33	*p* = 0.55			*p* = 0.10	*p* = 0.88
	W9				*p* = 0.73				*p* = 0.36				*p* = 0.17				*p* = 0.07				*p* = 0.71				*p* = 0.14

IC	BL	*p* = 0.01	*p* = 0.49	*p* = 0.51	*p* = 0.01	*p* = 0.77	*p* = 0.40	*p* = 0.00	*p* = 0.00	*p* = 0.00	*p* = 0.00	*p* = 0.00	*p* = 0.00	*p* = 0.61	*p* = 0.23	*p* = 0.02	*p* = 0.00	*p* = 0.01	*p* = 0.10	*p* = 0.68	*p* = 0.54	*p* = 0.58	*p* = 0.01	*p* = 0.01	*p* = 0.01
	W1		*p* = 0.01	*p* = 0.01	*p* = 0.01		*p* = 0.26	*p* = 0.00	*p* = 0.00		*p* = 0.58	*p* = 0.43	*p* = 0.04		*p* = 0.48	*p* = 0.00	*p* = 0.00		*p* = 0.38	*p* = 0.03	*p* = 0.00		*p* = 0.01	*p* = 0.01	*p* = 0.01
	W5			*p* = 0.18	*p* = 0.01			*p* = 0.00	*p* = 0.00			*p* = 0.81	*p* = 0.13			*p* = 0.00	*p* = 0.03			*p* = 0.22	*p* = 0.02			*p* = 0.24	*p* = 0.02
	W9				*p* = 0.01				*p* = 1.00				*p* = 0.20				*p* = 0.00				*p* = 0.31				*p* = 0.25

		***F* = 3.92, *p* = 0.01, df = 4**				***F* = 5.49, *p* = 0.01, df = 4**				***F* = 8.16, *p* = 0.00, df = 4**				***F* = 3.44, *p* = 0.02, df = 4**				***F* = 2.35 *p* = 0.05, df = 4**				***F* = 13.98, *p* = 0.01, df = 4**			

	BL	*p* = 0.31				*p* = 0.05				*p* = 0.01				*p* = 0.68				*p* = 0.55				*p* = 0.90			
IC	W1	*p* = 0.15				*p* = 0.12				*p* = 0.37				*p* = 0.52				*p* = 0.06				*p* = 0.56			
X	W5	*p* = 0.04				*p* = 0.86				*p* = 0.15				*p* = 0.46				*p* = 0.99				*p* = 0.01			
FC	W9	*p* = 0.09				*p* = 0.01				*p* = 0.74				*p* = 0.01				*p* = 0.56				*p* = 0.01			
	W13	*p* = 0.01				*p* = 0.02				*p* = 0.01				*p* = 0.78				*p* = 0.01				*p* = 0.01			

*General linear models (GLM) and Fisher’s *post hoc* tests were used to investigate the variations of behaviors, fecal cortisol, and body weight between groups (FC/IC) or sex (in DPT) throughout the study phases. Results were considered statistically significant at *p* ≤ 0.05 and 0.05 < *p* < 0.07 as statistically trend. All p values in black correspond to statistical significance and values in gray to non-significant ones*.

*BL, baseline; FC, familiar context; IC, isolated context; W1, Week 1; W5, Week 5; W9, Week 9; W13, Week 13*.

#### Behavioral Profile

No significant differences between groups nor variations across phases were observed in scent marking, individual piloerection and feeding [GLM test, Phases*Groups; scent marking: *F* = 0.77, *p* = 0.54, df = 4; individual piloerection: *F* = 2.03, *p* = 0.09, df = 4; feeding (frequency): *F* = 0.44, *p* = 0.77, df = 4]. However, during the IDS protocol, significantly higher scores for individual piloerection for the IC compared with the FC group were detected (GLM test, Group*; individual piloerection: *F* = 5.22, *p* = 0.02, df = 1).

Regarding locomotion, a significant increased occurred in W1 relative to BL, W9, and W13, but not to W5. In W13, the frequency of locomotion was similar to that of BL (Figure 3B). All statistical values are in Table 2.

In addition to IC, consecutive significant increases in the frequency of scratching were observed in W5, W9, and W13 (Figure 3C). During the IDS protocol, analyzing all weeks together, significantly higher scores for scratching were found for the IC group than in the FC group (GLM test, Group*; scratching: *F* = 25.55, *p* = 0.01, df = 1).

For autogrooming (duration in seconds), significant increases were observed for both groups, but those of the FC group occurred only between W13 vs. W1 and W5. For IC, significant increases in autogrooming behavior were detected at W9 and W13 relative to BL, W1 and W5. All statistical values are in Table 2. Although both conditions (familiar and socially isolated), showed increases in W13, the duration of autogrooming was higher in IC than in FC at W13 and W9, but was higher in FC than in IC at BL (Figure 3D). All statistical values are in Table 2.

We observed a significant reduction in the frequency of ingestion of sucrose solution in FC at W13 relative to BL and W5. For IC were observed reduction in initials and finals phases of isolation with respect to BL. All statistical values are in Table 2. Moreover, the percentage of reduction in the frequency of ingestion of sucrose solution, comparing BL and W13, was higher in IC than in FC (Mann–Whitney *U* = 1.00, *p* = 0.01), while IC reduced the intake by approximately 65.99%, and FC reduced it by 26.95% (Figure 3E).

For IC, correlation analysis along the experimental phases (W1, W5, W9, and W13) showed significant negative correlations between cortisol and autogrooming and scratching and positive correlations between locomotion and scent marking, individual piloerection, and scratching (Table 3). No significant statistical correlations were found in FC among the same comparisons.

**Table 3:** Significant *Spearman* correlation (*p* < 0.05), between behaviors, and behaviors versus cortisol, in both experimental protocols of the study, IDS (protocol for induction of depression state; IC, isolated group, males = 5) and DPT (protocol for depression state + pharmacological treatment; males = 8, females = 7).

IC group (IDS)	Males + females (DPT)
Cortisol*AutoG (*rs* =−0.16)	AutoG*Loc (*rs* =−0.15)
Cortisol*Scrat (*rs* = −0.19)	Scrat*Cortisol (*rs* =−0.21)
Loc*ScentM (*rs* = 0.46)	Scrat*AutoG (*rs* = 0.53)
Loc*PiloI (*rs* = 0.15)	
Loc*Scrat (*rs* = 0.22)	

*AutoG, autogrooming; PiloI, individual piloerection; ScentM, scent marking; Loc, locomotion; Scrat, scratching*.

### Body Weight

The body weight records over 13 weeks showed a parallel profile of weight gain in both groups, except for the IC group in W9, when the values decreased. Statistically significant differences between groups occurred at this point, where FC showed higher weight than IC. It is interesting to note that at W13, IC animals showed a recovery of weight and presented higher values than those observed in the initial phases, which was similar to that observed in the FC group (Figure 3F). All statistical values are in Table 2.

### Protocol for DPT: Predictive Validity IC

#### Hormonal Profile

**Figure 4:**
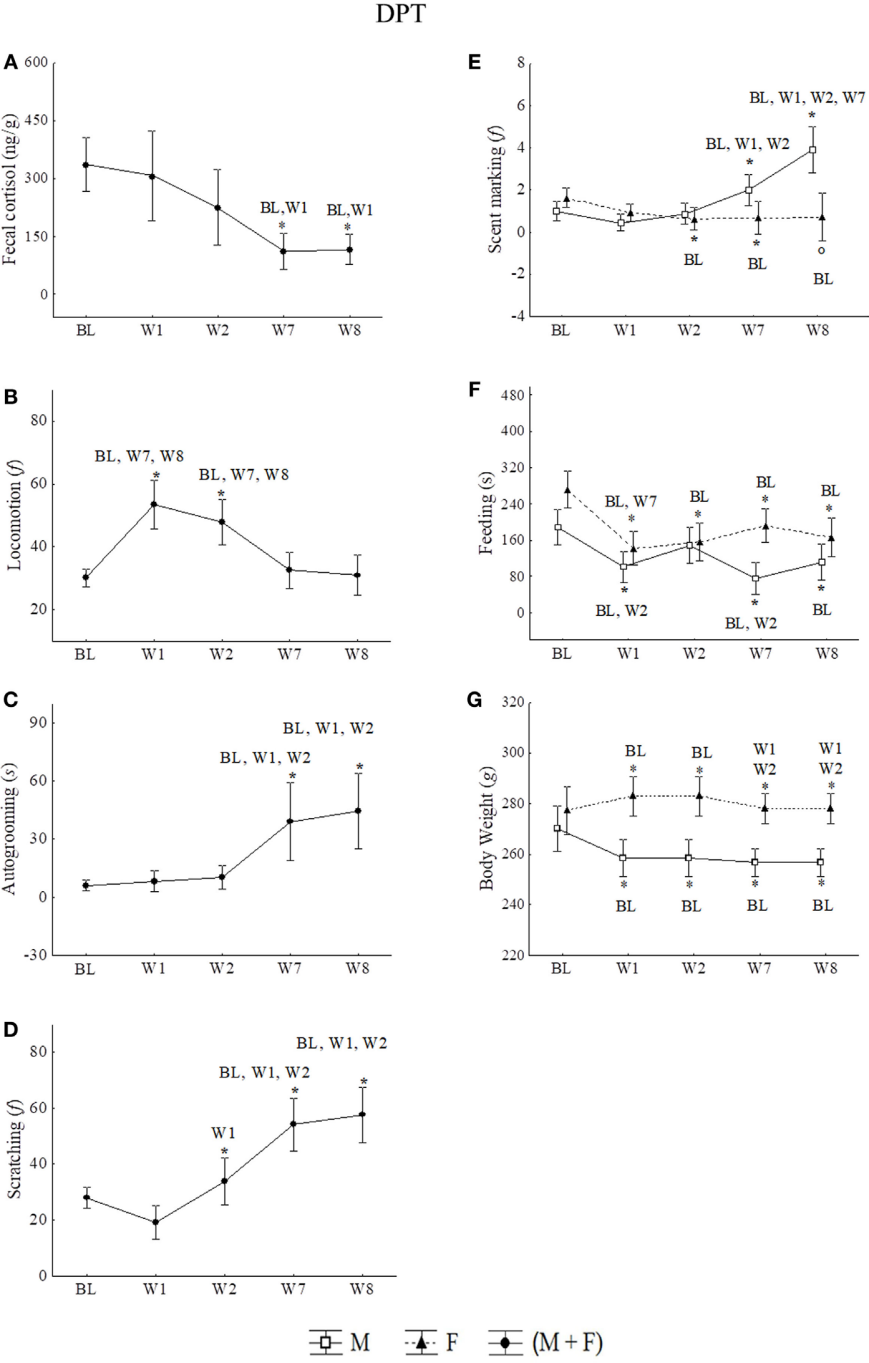
Mean of the week + SEM for **(A)** fecal cortisol, **(B)** locomotion, **(C)** autogrooming, **(D)** scratching, **(E)** scent marking, **(F)** feeding, and **(G)** body weight in male and females juveniles *Callithrix jacchus* along DPT (protocol for depression state + pharmacological treatment) in baseline (BL) and Isolated context (IC; W1, W2, W7, and W8). *Statistically significant (*p* ≤ 0.05) differences between the phase in which the symbol is and the (s) phase (s) indicated next to the symbol; °statistically trend (0.05 < *p* < 0.07) of difference between the phase in which the symbol is and the phase indicated next to the symbol. General linear models and *post hoc* Fisher.

In last 2 weeks of social isolation (W7 and W8), the cortisol levels of males (*n* = 8) and females (*n* = 7) decreased significantly below BL levels and W1 (Figure 4A). All statistical values are in Table 4.

**Table 4:** Statistical values of the protocol for depression state + pharmacological treatment (DPT): baseline (BL) + isolated context (IC).

	Fecal cortisol					Locomotion				Autogrooming				Scratching			
	*F* = 3.88, *p* = 0.00, df = 4					*F* = 14.05, *p* = 0.00, df = 4				*F* = 9.47, *p* = 0.00, df = 4				*F* = 22.77, *p* = 0.00, df = 4			
		W1	W2	W7	W8	W1	W2	W7	W8	W1	W2	W7	W8	W1	W2	W7	W8
M + F	BL	*p* = 0.44	*p* = 0.09	*p* = 0.00	*p* = 0.00	*p* = 0.00	*p* = 0.00	*p* = 0.54	*p* = 0.82	*p* = 0.80	*p* = 0.60	*p* = 0.00	*p* = 0.00	*p* = 0.08	*p* = 0.23	*p* = 0.00	*p* = 0.00
	W1		*p* = 0.36	*p* = 0.01	*p* = 0.01		*p* = 0.20	*p* = 0.00	*p* = 0.00		*p* = 0.78	*p* = 0.00	*p* = 0.00		*p* = 0.00	*p* = 0.00	*p* = 0.00
	W2			*p* = 0.14	*p* = 0.14			*p* = 0.00	*p* = 0.00			*p* = 0.00	*p* = 0.00			*p* = 0.00	*p* = 0.00
	W7				*p* = 0.99				*p* = 0.70				*p* = 0.55				*p* = 0.41

	**Scent marking**					**Feeding**				**Body weight**							
	***F* = 10.88, *p* = 0.00, df = 4**					***F* = 3.05, *p* = 0.01, df = 4**				***F* = 10.68, *p* = 0.00, df = 4**							
		**W1**	**W2**	**W7**	**W8**	**W1**	**W2**	**W7**	**W8**	**W1**	**W2**	**W7**	**W8**				

M	BL	*p* = 0.24	*p* = 0.79	*p* = 0.02	*p* = 0.00	*p* = 0.00	*p* = 0.08	*p* = 0.00	*p* = 0.00	*p* = 0.00	*p* = 0.00	*p* = 0.00	*p* = 0.00				
	W1		*p* = 0.36	*p* = 0.00	*p* = 0.00		*p* = 0.03	*p* = 0.26	*p* = 0.63		*p* = 1.00	*p* = 0.41	*p* = 0.41				
	W2			*p* = 0.01	*p* = 0.00			*p* = 0.00	*p* = 0.11			*p* = 0.41	*p* = 0.41				
	W7				*p* = 0.00				*p* = 0.11				*p* = 1.00				
F	BL	*p* = 0.14	*p* = 0.04	*p* = 0.05	*p* = 0.06	*p* = 0.00	*p* = 0.00	*p* = 0.00	*p* = 0.00	*p* = 0.01	*p* = 0.01	*p* = 0.75	*p* = 0.75				
	W1		*p* = 0.55	*p* = 0.64	*p* = 0.70		*p* = 0.55	*p* = 0.04	*p* = 0.30		*p* = 1.00	*p* = 0.03	*p* = 0.03				
	W2			*p* = 0.90	*p* = 0.83			*p* = 0.14	*p* = 0.65			*p* = 0.03	*p* = 0.03				
	W7				*p* = 0.93				*p* = 0.30				*p* = 1.00				

*General linear models (GLM) and Fisher’s *post hoc* tests were used to investigate the variations of behaviors, fecal cortisol, and body weight between groups (FC/IC) or sex (in DPT) throughout the study phases. Results were considered statistically significant at *p* ≤ 0.05 and 0.05 < *p* < 0.07 as statistically trend. All p values in black correspond to statistical significance and values in gray to non-significant ones*.

*M, males; F, females; BL, baseline; W1, Week 1; W2, Week 2; W7, Week 7; W8, Week 8*.

#### Behavioral Profile

No significant changes were observed in individual piloerection and somnolence across phases or by sex and between interactions of them (GLM test; individual piloerection, Phases*: *F* = 0.83, *p* = 0.50; Sex*Phase: *F* = 0.60, *p* = 0.65; somnolence, Phases*: *F* = 1.52, *p* = 0.19; Sex*Phase: *F* = 1.44, *p* = 0.21).

Males and females showed similar profiles of changes in autogrooming, scratching, and locomotion behaviors during social isolation phases. In the first 2 weeks (W1 and W2) of isolation, significant increases were observed in locomotion relative to the BL phase. Locomotion in W1 and W2 were also statistically higher when compared with the last 2 weeks (W7 and W8) when the frequency of displacements returned to BL values (Figure 4B). All statistical values are in Table 4.

Moreover, in both sexes, in W7 and W8, significant increases in autogrooming were observed with respect to all anterior phases (BL, W1, and W2) (Figure 4C). All statistical values are in Table 4. A weak negative correlation between autogrooming and locomotion was detected (Spearman correlation: *rs* = −0.15, *p* < 0.05) (Table 2).

Scratching behavior also presented a significant increasing pattern in W2, W7, and W8, as well as W2 with respect to W1, and W7 and W8 relative to BL, W1, and W2 (Figure 4D). All statistical values are in Table 4. A weak negative correlation between scratching and cortisol (Spearman correlation: *rs* = −0.12, *p* < 0.05) and a positive correlation between scratching and autogrooming in the IC group were observed (Spearman correlation: *rs* = 0.53, *p* < 0.05) (Table 3).

Scent marking and feeding behaviors showed a sexual dimorphic profile of variation during the social isolation period. Males increased scent marking behavior significantly in W7 and W8 with respect to anterior weeks. In contrast, females reduced this behavior in both W2 and W7 relative to the BL phase and showed a strong decreasing tendency in W8 relative to BL (Figure 4E). All statistical values are in Table 4.

Males showed a significant reduction in feeding (duration) at W1, W7, and W8 relative to the BL phase. In females, reduced feeding occurred during all phases of isolation in comparison with the BL phase (Figure 4F). All statistical values are in Table 4.

For males and females grouped together, the frequency and duration of ingestion of sucrose solution was significantly reduced between BL and W8 by 41.33 and 50.55%, respectively (Wilcoxon frequency: *z* = 3.14, *p* = 0.00; duration: *z* = 2.70, *p* = 0.00). Despite a large number of cases with zero frequencies, it was not possible to run a GLM test due to a high frequency of zeros.

#### Body Weight

Males and females showed a significant statistically dimorphic profile of alterations in body weight (GLM; Phases*Sex: *F* = 10.68, *p* = 0.00). Males experienced reduced body weight in the first week (W1) of isolation and maintained the reduction in all subsequent weeks of isolation (W2, W7, and W8). In contrast, females showed increased body weight at W1 and W2 with respect to BL. However, weight loss was observed at W7 and W8 with respect to W1 and W2 (Figure 4G). All statistical values are in Table 4.

### Vehicle Effect, PH, and tPE

#### Hormonal Profile

As described in the Methods section, from the 15 animals that were socially isolated (IC) (M = 8; *F* = 7), 3 males and 3 females were randomly selected to participate in a protocol of VE and pharmacological treatment (DPT) and were followed during 5 additional successive weeks after isolation (Week 8). In the first week (Week 9 = W9 = VE), common marmosets were subjected to a daily injection of VE (saline). In the 10th week (W10), a daily injection of antidepressant drug (nortriptyline chloride) was injected into the same animal (PH). Then, in the 11th, 12th, and 13th weeks (W11, W12, and W13) without any manipulation, all six animals were monitored for the tPEs. The data collected under these three conditions (VE, PH, and tPE) were compared with those obtained for the same animals in W8.

**Figure 5:**
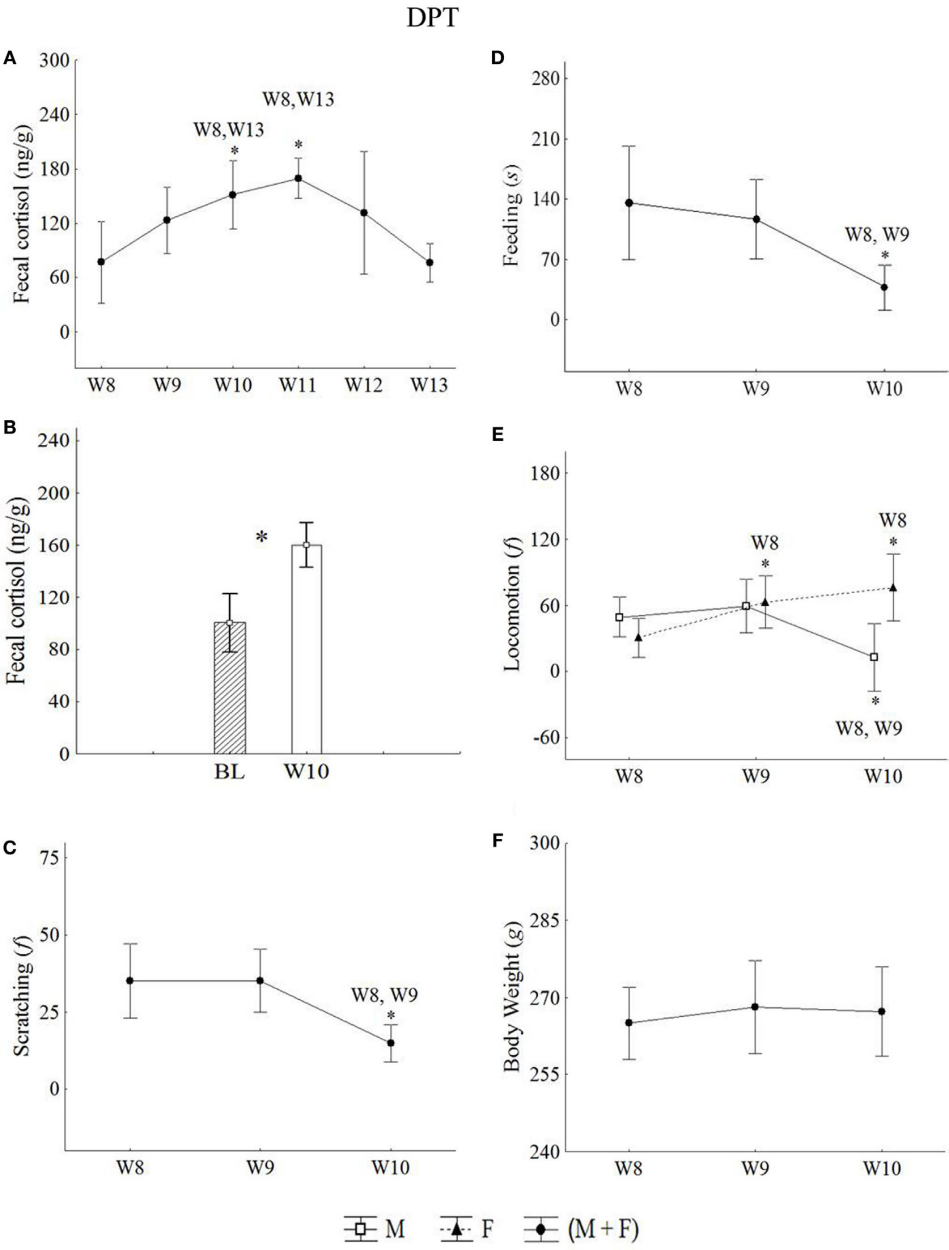
Mean of the week ± SEM for: **(A,B)** fecal cortisol, **(C)** scratching, **(D)** feeding, **(E)** locomotion, and **(F)** body weight in male and females juveniles *Callithrix jacchus* along DPT (protocol for depression state + pharmacological treatment) in baseline (BL) and isolated context (IC; W8), VE (W9), PH (W10), and tPE (W11, W12, and W13). *statistically significant (*p* ≤ 0.05) differences between the phase in which the symbol is and the (s) phase (s) indicated next to the symbol; °statistically trend (0.05 < *p* < 0.07) of difference between the phase in which the symbol is and the phase indicated next to the symbol. General linear models and *post hoc* Fisher.

Statistical increases in cortisol levels were detected in W10 and W11 relative to W8 (Figure 5A). All statistical values are in Table 5. The cortisol levels observed in the pharmacological treatment week (W10) was higher than BL levels (*t*-test = −2.80, *p* = 0.00) (Figure 5B).

**Table 5:** Protocol for depression state + pharmacological treatment (DPT): vehicle (VE), pharmacological (PH) + tardive pharmacological effects (tPE).

		Fecal cortisol					Scratching		Feeding	
		*F* = 3.16, *p* = 0.00, df = 5					*F* = 5.78, *p* = 0.00, df = 2		*F* = 6.63, *p* = 0.00, df = 2	
		W9	W10	W11	W12	W13	W9	W10	W9	W10
M + F	W8	*p* = 0.14	*p* = 0.00	*p* = 0.00	*p* = 0.07	*p* = 0.91	*p* = 0.94	*p* = 0.00	*p* = 0.52	*p* = 0.00
	W9		*p* = 0.23	*p* = 0.10	*p* = 0.72	*p* = 0.17		*p* = 0.00		*p* = 0.00
	W10			*p* = 0.66	*p* = 0.40	*p* = 0.01				
	W11				*p* = 0.20	*p* = 0.00				
	W12					*p* = 0.09				

		**Locomotion**								
		***F* = 8.87, *p* = 0.00, df = 2**								
		**W9**	**W10**							

M	W8	*p* = 0.48	*p* = 0.01							
	W9		*p* = 0.00							
F	W8	*p* = 0.02	*p* = 0.00							
	W9		*p* = 0.34							

*General linear models (GLM) and Fisher’s *post hoc* test were used to investigate the variations of behaviors, fecal cortisol, and body weight between groups (FC/IC) or sex (in DPT) throughout the study phases. Results were considered statistically significant at *p* ≤ 0.05 and 0.05 < *p* < 0.07 as statistically trend. All p values in black correspond to statistical significance and values in gray to non-significant ones*.

*M, males; F, females; W8, Week 1; W9, Week 9; W10, Week 10; W11, Week 11; W12, Week 12; W13, Week 13*.

#### Behavioral Profile

Males and females reduced significantly the scratching and feeding in response to pharmacological treatment (W10). This same reduction was not observed in the VE (W9) with respect to W8 (Figures 5C,D, respectively). All statistical values are in Table 5.

Females increased significantly the locomotion in the VE (W9) and pharmacological weeks (W10) with respect to W8, whereas males reduced locomotion in W10 with respect to W8 and W9 (Figure 5E). All statistical values are in Table 5.

Median somnolence in W8 and W9 were similar, μ = 0.00 ± 0.00. However, an increase in somnolence was observed in W10 with respect to W8 (μ = 3.77 ± 2.88) (Wilcoxon *z* = 2.36, *p* = 0.01).

Despite the low variability and large number of case with zero frequencies of sucrose solution ingestion, in W8, W9, and W10, once again, the GLM test and Wilcoxon test could not be conducted (median: W8, *p* = 0.00, W9, *p* = 0.00 and W10, *p* = 0.00).

#### Body Weight

Males and females did not present significant variations in body weight (GLM test; Phases*Sex: *F* = 0.17, *p* = 0.83, df = 2) during the VE and pharmacological treatments (Figure 5F).

## Discussion

The aim of this study was to validate *C. jacchus* as a translational juvenile animal model using chronic social isolation as a protocol inductor of depression, meeting the distinct criteria of validation: etiologic, face, functional, predictive, inter-relational, evolutionary, and population.

To meet the population criteria and mimic the variability observed in human populations, 25 common marmosets, males and females, were randomly selected from 20 different families of the Laboratory of Advanced Studies in Primates of the Universidade Federal do Rio Grande do Norte, Natal, Brazil. This laboratory had approximately 150 animals with fair genetic variability, living under captive conditions, and their pedigree comes from animals captured from nature and those born in captivity.

To address the etiologic criteria of validation, we elected to use a chronic social stressor protocol to induce the depression state in non-human primates, as social stressors seem to be the most prevalent inductors of depression in humans. Social species exhibit behavioral interactions with their conspecifics, who express adapted neural, hormonal, cellular, and genetic mechanisms that support survival, reproduction, and care of offspring. Consequently, social isolation causes disruptions in the social relationship and dysfunctions in physiological mechanisms reducing the adaptability and inducing the onset of physical and mental disorders (28, 60, 61).

To meet the face and functional domains of validation criteria, initially, 10 juvenile males were chronically socially isolated for 13 weeks, and modifications in their behaviors were investigated, including hormonal profile (fecal cortisol) and body like state. The alterations observed in socially isolated animals were not observed in body weight. We observed that this procedure triggered significant physiological and behavioral changes, and some of those showed intervariable correlations, which supported the investigated inter-relational criteria. Several of these alterations were similar to those observed in humans and/or in non-human primates in the depressive-like state. The alterations observed in socially isolated animals were not observed in common marmosets of the same sex and age that remained in their family environments.

Moreover, to address inter-relational and evolutionary criteria of validation, we analyzed species-specific behaviors such as individual piloerection, scent marking, scratching, and autogrooming, as well as behaviors that can be compared among species, such as locomotion, anhedonia, feeding, and somnolence. Indeed, body weight and cortisol levels were analyzed, constituting parameters that can also be used for comparison across species in other studies.

Our results showed that animals who remained in the family group (FC), in protocol 1 (IDS) exhibited no significant alterations in cortisol levels throughout the 13 weeks of the study. This is expected, since the social and environmental conditions were unchangeable and supportive. In accordance with the “theory of main effect,” social support is a buffer against the challenges posed by stressors of the daily routine, reducing stress responses and the onset of associated pathologies (62). In marmosets, social and affiliative behaviors such as allogrooming and body contact, induce reductions in cortisol levels (63). However, the benefits of social support are not a general phenomenon and depend on many factors such as species, sex, age, temperament, genetic relatedness, and familiarity (39, 64).

The changes in hyper and hypocortisolism are entirely linked to a variety of other alterations in common marmosets, showing their regulatory influence to face isolation from the family group. Hypercortisolemia was observed as an acute response, at the beginning of isolation in the animals and might promote changes similar to those observed in humans, such as hyperglycemia, increased cardiovascular and respiratory system, inhibition of the reproductive system and cellular growth, imbalance of the immune system, and a greater susceptibility to viral infections. While hypocortisolism was recorded during the chronic phase of isolation and can cause damage to the animal since it is associated with the expression of hypoglycemia and dysregulation of the immune system and greater propensity for bacterial infections. Moreover, it can trigger severe inflammatory processes, characterized by reduced energy in the animal and a state of apathy, characteristic of depression associated with chronicity (49).

By contrast, animals of the IC group displayed increased cortisol levels and an increased frequency of locomotion in the initial week of isolation (W1), characterizing a typical endocrine and behavioral anxious stress response to an acute stressor. Afterward, they displayed a reduction in both of these variables, indicating a recovery of the initial stress response. It is important to note that the random displacement of the animals after isolation observed in this study differs from the approximation and withdrawal directed to another animal or object being considered as an anxious behavior, because it occurs without a defined interest (65). These changes in locomotion were positively correlated with scent marking and individual piloerection behaviors, which are also considered anxious behaviors in a stress context (53, 54, 66). IC animals also exhibited higher levels of individual piloerection than those in FC during W1 to W13 of the IDS protocol. Likewise, individual piloerection also is considered an indicator of activation of the sympathetic nervous system (54).

The continued exposure to stressors induced other alterations in IC animals, showing that the ability to self-adjust allostatic systems fails. In subsequent phases, IC animals showed an increase in scratching from W5 to W13, which remained higher than that of FC in the IDS protocol. Additionally, a negative correlation between cortisol and scratching in IDS (W1 to W13) was observed. Scratching also occurs as dislocate behavior, without the cleaning function, and is also included as typical depression-like behavior in non-human primates (29, 65, 67). The occurrence of ethologically abnormal patterns where the animals express stereotypic behaviors and/or self-mutilation might indicate the low quality of life of animals and the presence of behavioral disorders (68, 69).

During the last two phases of IDS (W9 and W13), increases were registered in the duration of autogrooming, which is a reducer of tension behavior, for socially isolated animals. For the IC group, this increase in autogrooming correlated positively with a reduction of cortisol levels. Autogrooming reduces tension by induction of oxytocin release, an inhibitor of activation of the HPA axis (55, 56, 70). A similar result was recorded for *Callithrix geoffroyi* when an inverse association between the frequency of social grooming and urinary cortisol was demonstrated (63).

Anhedonia corresponds to an inability to feel pleasure, which is an important symptom in human patients with depression. In non-human primates and in rodents, it has been investigated by measuring the intake of an aqueous solution of sucrose (29, 57, 65, 71, 72). In this study, both groups decreased their consumption of sucrose solution during the 13 weeks of IDS, as expected, since consumption declines when a sweet taste is no longer a novelty. However, the profile of the reduction was different between the two groups, showing that the reduction in IC was faster than that in FC, taking place in W1 in IC and only starting in W13 in FC. Furthermore, the proportion of reduction of consumption of sucrose solution between basal and final ingestion was higher in IC than in FC, suggesting that IC animals developed an anhedonic state, similar to that of human patients with depression.

The IC animals reached the end of the study showing lower cortisol levels than those observed at BL and lower than those in FC. Studies in depressed human patients or in animal models of depression found conflicting results regarding cortisol levels (49, 73, 74). The majority of animal studies used rodents as models, for which protocols differ in nature and the duration of the stressor with respect to those used in this study (8, 29). Most protocols available in the literature involve acute situations and frequently use physical stressors known as “earned helplessness” (23, 75). Moreover, in protocols in which social stressors are used, such as early maternal separation and social defeat, the results should be evaluated with care because these stressors can have low ecological validity depending on the rodent species used (76, 77). It is important to consider that some rodents have complex social organization, such as free-living populations of house mice (*Mus musculus domesticus*), in which females display social cooperation such as regular nursing of non-offspring (76). However, some rodent species display solitary habits and lower parental care in their natural environment, as observed in montane (*Microtus montanus*) and meadow (*Microtus pennsylvanicus*) voles and African mole-rats (78–83). In these distinct taxa the neurobiology, behaviors, as well as reproductive and parental adaptations are at the opposite sides of the spectrum. Therefore, social stressors may have low ecological validity to taxa that adopt a strictly solitary lifestyle and lack the ability to form long-term social bonds.

However, recent studies that induced depression in non-human primates using paradigms of chronic social stressors such as rank dispute or social isolation, similar to that used in our study, found hypocortisolemia in response to these protocols. An association between low levels of cortisol and depressive-like behaviors in adult females of *Macaca fascicularis* exposed to chronic changes in social position (22–26 months) was demonstrated by Willard and Shively (49). For squirrel monkey (*Saimiri sciureus*) juveniles, subjected to repeated separation from their mothers as infants revealed cortisol hyporesponsiveness in adulthood when compared with a control group (84). Juvenile common marmosets that were exposed to repeated separation of their families in infancy present lower basal cortisol levels than animals of the same age that were not exposed to this stressor (54).

In humans while major depression is traditionally characterized by hypercortisolemia (85), specific groups have been showing hypocortisolemia, like patients with atypical subtype major depression, patients with maladaptive coping styles and major depression associated with severe, refractory and chronic conditions (86–90). Currently, two theories attempt to explain the etiology of hypocortisolemia. The first associates hypocortisolemia with increased sensitivity of negative feedback on the HPA (91), and the second is linked to an adrenal insufficiency (92). Generally, the literature associates depression with a hyperactivation of the HPA axis and hypercortisolemia in depressed human patients or in animal models of depression (28, 73, 74).

Some studies have suggested that hypocortisolemia initially might be a protective adaptation of the individual after abrupt rises in cortisol in response to chronic stressful events (93). However, if this condition remains for an extended period, the ability to self-adjust allostatic systems fails, and pathologies arise (94). Therefore, in this study, one allostatic system that could likely have induced a severe reduction of cortisol in IC animals to pathological levels after W9 and fail to return cortisol levels might be autogrooming, as suggested by the negative correlation between cortisol and autogrooming recorded for IC.

The failure of allostatic systems to self-adjust might induce permanent physiological vulnerability, especially if it occurs during biological windows of plasticity, such as adolescence, therefore facilitating the subsequent emergence of psychiatric disorders such as depression (95, 96). Some studies show that when the first signs of depression arise during adolescence, those symptoms frequently will also be manifested in adulthood (97, 98).

In addition to alterations in behaviors and cortisol levels, during W9, a severe reduction in body weight of the IC group, but not in FC, was found in this study. The weight loss seems not to be related to a reduction in feeding, because the isolated animals did not alter the amount of food ingested during the IDS protocol. After a reduction of body weight in W9, the isolated animals recovered in W13, showing higher values relative to the BL phase. Loss of weight is a typical response of non-human primate adults subjected to depression protocols (29, 65). Nevertheless, the evaluation of this parameter during ontogenetic phases of rapid growth, such as the juvenile stage, might mask any results and generate false conclusions (99). Moreover, the relation between chronic stress and feeding appears to be quite individualized and studies with animal models suggest that stressful situations can lead to increases in some situations, but in others, it leads to decreases in feeding (100). In humans, similarly to animal models, stressful situations and depressive disorders can also act in both directions on feeding (101).

In summary, the animals of IC showed correlated behaviors and physiological (cortisol fecal and body weight) alterations, which included hypocortisolemia, high levels of scratching, and anhedonia, composing a typical depression-like state that is similar to alterations observed in other species of non-human primates or/and humans. Therefore, the results exhibited for common marmosets corroborate the validation of face, functional, inter-relational, and evolutionary criteria for this animal model.

After the above validation, to address predictive criteria of validation and to investigate sexual dimorphism in this model, eight more males and seven female juvenile common marmosets were randomly selected from different families to be socially isolated by 8 weeks and to subsequently be treated sequentially with a VE and nortriptyline (M = 3 and F = 3). The same parameters of the IDS protocol were investigated namely, behaviors, fecal cortisol, and body weight.

In large part, the alterations observed in protocol for DPT were similar between the sexes, with the exception of scent-marking and body weight. In common marmosets, puberty starts around 6–7 months, whereas sexual maturity is achieved at approximately at 16 months (35, 102). As a result, sexual maturity transitions of the animals used in this study may have produced variability in terms of sexual dimorphism of stress response, with some and not others being sexually dimorphic.

In the initial isolation phases of IC (W1 and W2), as expected, increases in locomotion and anxious behavior were observed in both sexes, similarly to the results observed in IDS protocols for inducing a depression-like state. Afterward, males and females reduced their cortisol production in the two final phases of isolation (W7 and W8), again consistent with the results of IDS. For common marmosets at this age, de Sousa et al. (40) found similar cortisol responses between male and female juveniles (12 months) after social isolation (21 days), in which they observed a drop in the cortisol response. Once again, probably, in DPT, autogrooming behavior acted as a decompensated allostatic mechanism responsible for the pathological reduction of cortisol, as both males and females showed an increase in the duration of autogrooming concomitant with decreased cortisol.

Also in W7 and W8, both sexes showed similar increases in the frequency of scratching, a stereotypical behavior. Moreover, the presence of anhedonia was inferred in W8; the animals reduced the frequency of ingestion of sucrose solution by 41%, both findings also analogous to those of the IDS protocol. By contrast, males and females showed dimorphic alterations in scent marking. Whereas males increased this behavior in the last two weeks of social isolation, females reduced scent marking between W2 and W8.

Moreover, males and females showed a reduction in feeding during IC and consequently, reductions in body weight, but distinct gradations between the sexes were observed. For males, weight loss was observed immediately (W1) and was sustained until W8. On the other hand, females initially gained weight and only lost weight in the last two phases of isolation (W7 and W8). These results in females corroborate the discussion presented for animals of the IDS protocol, which indicate difficulties in the analysis of body weight in this phase of ontogenetic development, because of intense growth and large individual variability.

Similarly and more consistent than the results of IC (IDS protocol), physiological and behavioral alterations observed in IC (DPT protocol) such as hypocortisolemia, high levels of scratching, and anhedonia associated with a reduction in feeding and body weight were typical of a depression-like state in non-human primates, allowing us to investigate the action of a traditional antidepressant drug in these animals, nortriptyline.

To address this, after the isolation phase (IC) of DPT protocol, three males and three females were randomly selected to be treated for 7 days (W9) with a VE followed by nortriptyline treatment for an equal period (W10). During W10, an increase was observed in cortisol levels above the values observed during BL, which was not induced by VE. This cortisol increase induced by drug was sustained for 7 additional days in W11 after suspension of the treatment. Subsequently, in W12 and W13, cortisol returned to similar levels to those observed in the final week of social isolation (W8).

The high cortisol levels detected in W10 might be related to the dose of nortriptyline and the duration of the pharmacological protocol used for marmosets. The dose used was the most effective (30 mg/kg ip) for rats in Moura (50), but our protocol was more extensive than that used in their study. Based on these findings, future protocols are needed that include different treatment dosages and/or durations for more adjusted changes.

Furthermore, after nortriptyline, but not with a VE, males and females exhibited reduced frequencies of scratching, reversing the high levels induced by isolation. This observation is very important, as scratching is a stereotypical behavior permanently observed in non-human primates and associated with a depression-like state (29, 65, 67).

Different profiles were observed for locomotive behavior in the sexes. Whereas females showed an increase in locomotion after both treatments using VE and nortriptyline, males reduced locomotion only with nortriptyline use. This evidence of a sexual dimorphic response shows the importance of using both males and females in protocols involving antidepressants drugs. For instance, in humans, different responses are observed between the sexes after the use of antidepressants (103).

An increase in somnolence and a reduction in feeding during the pharmacological phase, but not the VE phase, were observed in marmosets. These alterations can be considered as side effects of drugs that frequently induce sedation and loss of appetite in humans (104). Nevertheless, these results corroborate the validation of predictive criteria in our model, since nortriptyline reversed the hormonal and behavioral expressions of depression-like changes exemplified by cortisol and scratching levels, respectively.

In summary, both physiological and behavioral alterations found for male and female juvenile common marmosets in response to chronic social isolation can be characterized as a depressive-like condition, which was, in large part, reversed by nortriptyline. Thus, the present study supports the validation of *C. jacchus* as one potential translational model of juvenile depression. Additionally, the study demonstrated that social isolation is an efficient paradigm that works as an inducer of depression in this model. We have provided important evidence that this model and protocol meet all validation criteria such as etiologic, face, functional, predictive, inter-relational, evolutionary, and population, which allow its use in complementary areas of investigations, including neurochemistry studies to disclose new biomarkers as well as the development of new antidepressant drugs, such as those derived from natural compounds, which could be more effective in alleviating depression symptoms in humans.

## Ethics Statement

The animals were housed according to IBAMA (Brazilian Institute of Environment and Renewable Natural Resources) guidelines (Normative Instruction no. 169 of February 20, 2008), and the care standards for animals established by CONCEA—National Council for Animal Experimentation Control, Law No. 11.794 (October 8, 2008). In addition, the laboratory complies with international standards for *ex situ* maintenance of animals as defined by the Animal Behavior Society and the International Primatological Society. The study and experimental procedures were approved by the Animal Research Ethics Committee (UFRN protocol No. 019/2013 and protocol No. 034/2014).

## Author Contributions

NG-C and MS designed the experiments; FS and AG collected the experimental data, carried out statistical analysis, and prepared figures. NG-C, MS, FS, and AG prepared the manuscript.

## Conflict of Interest Statement

This research was conducted in the absence of any commercial or financial relationships that could be construed as a potential conflict of interest.

## Abbreviations

IDS, induction of depression state; BL, baseline; FC, familiar context; IC, isolated context; DPT: depression state + pharmacological treatment; PH, pharmacological treatment; VE, vehicle; tPEs, tardive pharmacological effects; AutoG, autogrooming; Loc, locomotion; PiloI, individual piloerection; ScentM, scent marking; scrat, scratching; W1, Week 1; W10, Week 10; W11, Week 11; W12, Week 12; W13, Week 13; W2, Week 2; W5, Week 5; W7, Week 7; W8, Week 8; W9, Week 9.
